# Social Protection in Health Care and Vulnerable Population Groups in Serbia

**DOI:** 10.3389/fpubh.2015.00194

**Published:** 2015-08-13

**Authors:** Jelena Arsenijevic, Milena Pavlova, Wim Groot

**Affiliations:** ^1^Department of Health Services Research, Faculty of Health, Medicine and Life Sciences, School for Public Health and Primary Care – CAPHRI, Maastricht University Medical Center, Maastricht University, Maastricht, Netherlands; ^2^Top Institute Evidence-Based Education Research (TIER), Maastricht University, Maastricht, Netherlands

**Keywords:** health, Serbia, vulnerable population groups, social protection, out-of-pocket patient payments

## Social Protection and the Health Care System in Serbia

Social protection refers to a set of policy measures to protect individuals, especially the critically poor, from financial losses due to high-risk events, such as natural disasters, social risks like unemployment, war or unexpected financial shocks, and political risks like discrimination of minorities in conflict zones (1–3). In terms of health, social protection includes protection against health risks, to ensure good quality of care and financial protection that aims to protect people from unexpected health care shocks (4). One way to assure financial protection in the health care sector is to introduce universal social health insurance (5). However, when universal health insurance cannot provide financial sustainability of the health care system, patient charges are necessary. With patient payments social protection can be achieved by the implementation of an exemption mechanism (5). In this paper, we focus on financial protection in health care in Serbia.

Serbia is a middle income country with long-term tradition in social protection related to health, inherited from the period of the former Yugoslavia (6–11). The health care system of Yugoslavia was known as a Swedish model in the Balkan (12). However, during the period 1991–2000, Serbia faced a civil war combined with a severe economic crisis (13). The crisis was followed by impoverishment among the citizens and the collapse of the existing health care system. Impoverishment was not the only consequence of the civil war. Like in many other post-conflict and transitional societies, corruption became a *modus vivendi* in the public sector in Serbia (14). The widespread corruption had a direct effect on health care consumers as well. Different types of informal (under the table) patient payments become common practice in the health care system (15).

After a major political change in 2000, the Serbian government introduced health care reforms. The main objective was to improve efficiency, service quality, and equity in health care (16). As part of the health care reforms, in 2002, the Serbian government introduced official co-payments for services covered by the compulsory health insurance to improve the financial situation of the public health care system. The introduction of official co-payments was accompanied with an exemption mechanism (7, 17).

## To What Extent does the Exemption Mechanism Protect Vulnerable Groups?

In Serbia, various population groups are exempted from paying for health care: children younger than 15 years, pregnant women, persons older than 65 years, disabled persons, HIV-infected persons, monks, people with low-family income, unemployed, chronically ill people, military service servants, people registered as refugees, and the Roma population (6). The government motivated the high number of exempted population groups by arguing that it reflected a long tradition of solidarity and equity in Serbia (18). However, recent studies show that the exemption mechanisms in Serbia are not effective (19). Two main reasons for the failure of exemption mechanisms are the design of the exemption mechanisms and the implementation of the exemption mechanisms. People with low income, although exempted, often report high amounts of payments (19). They are usually unaware of their rights. On the other side, some population groups with chronic conditions are not exempted or just partially exempted and they experience the burden provoked by patient payments. The implementation of the exemption mechanism also has weaknesses. Since the guidelines are not clear and written in law-centered language, it is very confusing for patients and health care providers to understand for which services the (partial) exemption mechanisms should be applied (20). Another obstacle in the implementation of the exemption mechanism is related to the procedure to obtain the exempted status. The procedure is administratively difficult and time consuming (21). At the same time, some population groups, even if they are not in need, are still exempted. These include military civil servants and monks, all people older than 65, and all children younger than 15. Those subpopulation groups were exempted during the civil war when the government of Slobodan Milosevic used the health care system as a political tool to buy social peace (22). However, not all people older than 65 and not all children younger than 15 are in financial need to be exempted. The payments for health care services for children are made by their parents. Not all parents are unable to pay for health care services. A better policy approach would be to use parents income as an indicator for exemption, instead of exempting all children younger than 15. Those examples confirm that the high number of exempted groups influence the financial sustainability of the health care system in Serbia. In the period 2003–2008, there were some attempts to reduce the number of health services that were included in the insurance package, but there was no attempt to decrease the number of exempted groups. During the parliamentary elections in 2004, 2008, and 2012, the main political parties emphasized that health care should remain free of charge for vulnerable population groups (23). The attempts of the previous Minister of Economy to simplify the exemption procedure and to decrease the number of exempted individuals led to his resignation (24). The Serbian case shows that when exemption mechanisms are not well designed and well implemented, vulnerable population groups may stay unprotected.

## To What Extent are Pregnant Women Protected within the Health Care System in Serbia?

In Serbia, maternity care is formally free of charge. All Serbian governments from 1991 to 2014 wanted to encourage women to have more children (25). In a country which is in a difficult politic and economy situation, a full exemption of pregnant women in maternity wards from official co-payments was one of the first financial protection measures taken. Financial protection also included prenatal and postnatal health services. Although exempted from official co-payments, many women report informal patient payments and quasi-formal patient payments (official charges set by the facility but not regulated by the government) (26). Quasi-formal payments are charged by hospitals for services that should be provided for free (e.g., epidural analgesia). Regarding the informal payments, the main reason for paying informally is to obtain better quality of care and safety for the new born child (27). However, recent studies show that informal patient payments do not guarantee better quality of care (26). Even though some women report informal patient payments, they still experience inconveniences related to quality of care, namely, problems with equipment and obligatory but non-necessary procedures during the admission. They also report poor bedside manners and derogative communications from the side of medical staff (26). Those inconveniences are related to psychological and social accessibility to health services and violate the official social protection measures. In order to avoid such inconveniences many women call upon “special connections.” “Special connections” are described as friends or relatives who work in the hospital and who can ensure a special treatment and adequate care. “Special connections” represent someone whom the pregnant woman can trust (26). In a certain way, they also represent a non-monetary way to buy compassion during the process of delivery. In this way, special connections represent a type of informal social protection. It means that pregnant women in Serbia are aware that formal social protection will not ensure adequate care in maternity wards. The existence of “special connections” also emphasizes that formal financial protection in Serbia is a necessary but not sufficient way to ensure adequate care.

## Perspectives for the Future

The examples presented above show that an adequate social protection policy for vulnerable population groups in health care should be based on different dimensions. Financial protection is one of these dimensions. Exemption mechanisms as a tool to achieve financial protection should be clearly designed and available for patients. A broad scope of exempted groups does not guarantee that the social protection policy is adequate and effective, especially in countries where the financial sustainability of health care systems is fragile. Instead of including a large number of population groups (such as in Serbia), attention should be paid to the adequate targeting of eligible groups (for example, using the already existing insurance system) and the adequate provision of health care services for those who are exempted. A good social protection policy should not only be more pro-poor oriented but also take in account health status. For example, the current policy could emphasize the importance of prevention of chronic diseases (28). People who are already diagnosed with a chronic disease are faced with the financial burden of using health care. Also, the example of maternity wards in Serbia shows that financial protection is a precursor but it is not enough to secure adequate social protection. Quality of care is another dimension of an adequate social protection policy. Within the maternity wards in Serbia, a lot is still left to be desired regarding the quality of care provided (29–32). Although recent studies show that physician’s skills are not perceived as bad, their communication skills are (26). In order to provide good services in maternity wards, the Serbian government should take women’s preferences into account. The government should also educate physicians to respect women’s preferences. This means that good physicians’ skills are a necessary but not a sufficient condition for providing good quality care (31, 33). The current system of medical education in Serbia is mainly focused on the technical skills of future physicians. Patients’ needs are not recognized as important for the curative process. Physicians in Serbia need to become aware that the satisfaction of patient needs contributes to more effective curative outcomes (34).

Despite the efforts of policy makers in Serbia to provide generous social protection policy, vulnerable population groups are still non-protected. Serbia shows that social protection policy in countries with limited financial resources should not be overgenerous. An adequate policy should focus on better targeting of those in need. Furthermore, social protection policy is related to official co-payments neglecting the existence of informal patient payments. More attention should be paid to prevent informal and quasi-informal patient payments. Also, future policy should recognize the importance of patient preferences.

## Conflict of Interest Statement

The authors declare that the research was conducted in the absence of any commercial or financial relationships that could be construed as a potential conflict of interest.
